# Comparing Breast Screening Protocols: Inserting Catch Trials Does Not Improve Sensitivity over Double Screening

**DOI:** 10.1371/journal.pone.0163928

**Published:** 2016-10-10

**Authors:** Weijia Chen, Piers D. L. Howe

**Affiliations:** School of Psychological Sciences, University of Melbourne, Parkville, Victoria, Australia; Monash University, AUSTRALIA

## Abstract

Breast screening is an important tool for the early detection of breast cancers. However, tumours are typically present in less than 1% of mammograms. This low prevalence could cause radiologists to detect fewer tumours than they otherwise would, an issue known as the prevalence effect. The aim of our study was to investigate a novel breast screening protocol, designed to decrease the number of tumours missed by radiologists, without increasing their workload. We ran two laboratory-based experiments to assess the degree to which the novel protocol, called the catch trial (CT) protocol, resulted in greater sensitivity (d’) than the double screener protocol (DS), currently utilised in Australia. In our first experiment we found evidence that the CT protocol resulted in a criterion shift relative to the DS protocol but the evidence that sensitivity was greater in the CT protocol relative to the DS protocol was less clear. A second experiment, using more realistic stimuli that were more representative of actual tumours, also failed to find convincing evidence that sensitivity was greater in the CT protocol than in the DS protocol. This experiment instead found that both the hit rate and the false alarm rate increased in the CT protocol relative to the DS protocol. So while there was again evidence that the CT protocol induced a criterion shift, the sensitivity appeared to be approximately the same in both protocols. Our results suggest the CT protocol is unlikely to result in an improvement in sensitivity over the DS protocol, so we cannot recommend that it be trialled in a clinical setting.

## Introduction

Visual search is a vital part of many professions such as cancer screening and airport baggage screening. In these fields missing a target could have severe consequences. Unfortunately, the proportion of trials on which a target is present (referred to as the target *prevalence* rate) in these professional searches is often very low. For example, the incidence rate for breast cancer is approximately 115 per 100,000 cases in Australia, i.e. a mere 0.115%[1]. When the target rarely appears, miss rate (i.e. the proportion of trials where the target is present but the radiologist responds that it is absent) increases dramatically compared to when the target is common (e.g. [2–6]), an issue known as the *prevalence effect* [6]. In an artificial baggage screening task, decreasing the proportion of target-present trials from 50% to 1% increased the miss rate four-fold [6]. Similar results have been observed using classical visual search stimuli, such as searching for a T among Ls [4, 7]. The prevalence effect is very robust and even trained radiologists [8, 9] and transportation security officers [10] are not immune from it.

In some circumstances, the prevalence effect can be attributed to observers developing a motor response bias [11]. This response bias decreases the time taken to make target-absent responses at the potential cost of the observers reporting too quickly that the target is absent and thus missing the target when it is in fact present [2, 4–7, 12, 13]. This motor response bias can be eliminated by offering observers the option to correct their responses [11], or by enforcing a delay before the observers can respond [4]. While motor response errors accounted for elevated miss rates at low prevalence in feature search or simple conjunctive search tasks with a small set size [4, 12], for more difficult search tasks, the prevalence effect could not be mitigated by an option to correct one’s former response or by forcing observers to confirm their responses, indicating a different cause for the prevalence effect in these circumstances [14].

In the terms of signal detection theory [15], sensitivity (*d’*) denotes how easily or difficult the target can be distinguished from the noisy background and criterion (*C*) reflects an observer’s bias towards reporting that the target is present. While there are conflicting accounts as to whether the target prevalence rate affects sensitivity [5, 9, 10], it has been consistently shown that the prevalence rate does cause the decision criterion to shift [4–6, 12, 14, 16]. As the prevalence rate decreased, these studies found that the criterion became more “conservative”; observers were biased towards reporting the target as absent, so were more likely to miss targets. Conversely, high prevalence lead to a more “liberal” criterion where searchers were biased towards reporting the target as present, so were more likely to make “false positives”, reporting that the target was present when it was in fact absent [10, 12, 17].

The prevalence effect is a stubborn source of miss errors that cannot be easily eliminated [5]. Having two observers search through the same set of images does not reduce the miss rate below that of the lower of the two [5]. Presenting half of the search image or the stimulus set first then followed by the other half to encourage a more thorough search does not mitigate the prevalence effect [12]. Moreover, simply mandating longer search times in a difficult search task does not reduce miss errors either [5].

Introducing decoy targets to boost the overall target prevalence rate did reduce the prevalence effect, as long the decoy belonged to the same category as the target [5]. For example, when the target was “any tool”, hit rate for a rare tool improved when another tool was frequently presented. But when the decoy and the target belonged to different categories this effect did not occur: a water bottle that appeared on 44% of the trials did not reduce the miss rate for a gun that appeared only on 1% of the trials [5].

Wolfe and colleagues [5] also trialled another approach to reducing the prevalence effect, which they found to be more effective. In this approach, they inserted “bursts” of high prevalence trials into a low prevalence search task. In an X-ray luggage screening task participants were shown 300 training trials with a target prevalence of 50%. They were then tested on 1,000 trials at 2% prevalence with no feedback. Among these low prevalence trials, the authors inserted 10 blocks of 40 trials where the target prevalence was 50%. In these high prevalence blocks, participants were provided with feedback. Results showed equivalent performance for both the 2% and the 50% prevalence trials. In particular, the miss rate was not elevated in the low prevalence trials. The authors suggested that the insertion of high prevalence “bursts” allowed observers to maintain a high prevalence criterion in the low prevalence condition so that their decision criterion remained about the same throughout the entire task [5]. While this decreased the number of misses, it also increased the number of false positives, causing d’ to remain approximately the same.

This burst approach was adopted by two further studies [9, 10]. Evans et al. investigated to what degree the burst approach could influence cytologists error rates in screening for cervical cancer. Conversely, Wolfe et al. [10]investigate to what degree the burst approach could influence the error rates of baggage screeners. Similar to Wolfe et al. [5] both studies found that a burst of high prevalence trials could induce a more liberal criterion, both within the block of high prevalence trials but also in the following low prevalence trials. However, contrary to Wolfe et al. [5], both studies found that a burst of high prevalence also increased sensitivity (d’).

From these studies, it is clear that the burst approach is effective at inducing a more liberal criterion. However, it seems not to also always improve sensitivity. The reason for this appears to be that in some studies the prevalence effect does not affect d’, so reducing the prevalence effect does not improve d’. In particular, for the stimuli used in Wolfe et al. [5], d’ was not affected by prevalence, but for the stimuli used in Evans et al. [9] and Wolfe et al. [10]d’ was. In this paper we are concerned with how the prevalence effect affects the screening of mammograms. For this sort of stimulus, based on the data reported by Evans et al. [8], it would appear that lowering the prevalence lowers d’. On this basis, we would expect the burst approach to increase d’ for our stimuli.

However, we can probably do better than the burst approach. The problem with the burst approach is that the effects of the high prevalence block wear off relatively quickly, over the course of a few dozen trials [12, 16]. In our project we therefore adopted a different approach, where we kept the prevalence rate high for the entire condition by inserting a large number of additional trials where the target was always present.

Breast Screen Victoria is one of the major readers of mammograms in Victoria, Australia. They are aware that radiologists sometimes miss potential cancers in mammograms and in an attempt to minimise this problem they have instituted a double screener (DS) protocol whereby each mammogram is initially viewed by two radiologists (the *observers*). If both radiologists reach the same conclusion it is recorded as final; if the two radiologists disagree, a third radiologist (the *reviewer*) is called in to view the image. Obviously this method is highly resource intensive and it is unclear how much it really reduces miss errors. Wolfe et al. [5] have previously shown that double reading is a particularly poor strategy for reducing the prevalence effect–if one searcher misses the target, the other one tends to as well.

The aim of our study was to develop a better protocol for breast screening: one that increased sensitivity without increasing workload. In this new protocol we arranged for each display to be viewed by only one observer. This halved the number of displays that each observer needed to view, allowing us to introduce a large number of specially-created additional displays that always contained a target. We could give feedback on these displays since we knew that they always contained a target. From the point of view of each observer, this greatly increased the target prevalence rate. We shall call this the catch trial (CT) protocol. Our aim was to compare d’ in the CT protocol to d’ in the double screener (DS) protocol. We expected d’ to be larger in the CT protocol than in the DS protocol, although the workload would be the same in both protocols. Our original intention was to demonstrate that the CT protocol was superior to the DS protocol in a laboratory setting and on the basis of this evidence arrange for a large-scale clinical trial. Unfortunately, as discussed below, we were unable to demonstrate that the CT protocol was superior to the DS protocol.

## Experiment 1: Ts and Ls

In our first test of the effectiveness of the catch trial (CT) protocol, we opted to use stimuli that had been used in a large number of previous visual search experiments, including some of those that had shown the prevalence effect (e.g. [4, 7, 16, 18]). This ensured that the properties of the stimuli were well understood. In our experiment, observers were asked to search for a rotated letter “T”. The T was not present on all the trials and occasionally a rotated “L” would be presented instead. The L was constructed so that it looked similar to a rotated T, so could be readily mistaken for one.

### Method

This study was approved by The University of Melbourne Human Research Ethics Committee (Ethics ID 1339889.2). All participants provided informed written consent.

#### Participants

Thirty-four participants between the ages of 19 and 28 years participated in this experiment (*M* age = 23.0 years, *SD* = 2.4 years; 22 women). Among them 14 participants were paired to form 7 pairs of initial observers in the DS condition, and 13 of these observers were also tested in the CT condition. The other 20 participants were recruited as reviewers. All 20 reviewers participated in both the DS and CT conditions. All participants had normal or corrected-to-normal visual acuity (at least 20/25; near-field Snellen eye chart) and normal colour vision (Ishihara plates). All participants gave informed consent and each were paid $15/hour for their participation.

#### Materials

**Fig 1:**
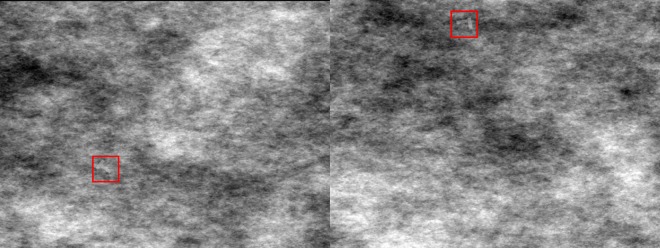
Sample stimulus of a target trial (T, left) and a distractor trial (L, right) from Experiment 1.

The stimuli comprised a noise background (different on each trial) onto which in some of the trials a white semi-transparent (opacity = 30%, luminance = 33.2 cd/m^2^) rotated letter was superimposed. This letter could be a T or an L subtending 0.57° x 0.76° of visual angle. The stem of the L was offset 0.095° of visual angle relative to its crossbar to make it look more similar to a T. The Ts were the targets and the Ls were the distractors. The stroke width was 0.095° of visual angle and the viewing distance was 60 cm. The sequence at which these background images were presented, and the location of the letter stimuli were randomised for each observer in the CT condition, and for each pair of observers in the DS condition. Fig 1 shows an example of the stimulus used in this experiment.

#### Procedure

The stimuli were presented on a personal computer using MATLAB^®^ and the Psychophysics Toolbox [19, 20] subtending an area of 36.0°×25.4° at the 60-cm viewing distance. There were two conditions in this study.

DS Condition. Seven pairs of observers searched through 7 sets of images for a single letter T rotated to any orientation, each observer searching independently. The prevalence rate for this target was 4% (i.e. a T was present on 4% of the trials). A distractor L was present on another 4% of the trials and observers were instructed to ignore it. The remaining trials contained neither the target nor the distractor. The distractors simulated benign tumours while the targets represented malignant tumours. The stimulus remained on screen until the observer responded “target present” by clicking on the target with the mouse, or by indicating that the target was not there by clicking on the words “target absent” at the top of the screen. They were then asked to confirm their response and were provided with the option to return to the search image and re-enter their response if they made a mistake. In this way we minimised any potential motor response errors [11, 14]. The first 50 trials were used as practice trials and feedback on response accuracy was given on each trial. No feedback was given for the rest of the trials. The reviewers were presented with those trials on which one member of the pair of observers responded “target present” while the other responded “target absent”, or when both observers responded “target present” on the same trial but disagreed on the location of the target. The reviewer judged whether the area(s) ringed by the initial observers contained a target. No feedback was provided either to the initial observers or to the reviewers.

CT Condition. A new set of images was created in the CT condition. These were created in the same way as in the previous condition, so were equivalent to the images in the previous condition. We shall call these the test images. As with the images in the DS condition, the target (a rotated T) appeared on 4% of the trials and the distractor (a rotated L) appeared on an additional 4% of the trials. Unlike in the previous condition where each of these images was initially viewed by a pair of observers, in the CT condition each of these test images was initially viewed by only one observer, thereby halving the number of test images that each observer needed to view. This allowed us to introduce a large number of additional images for each observer. These additional images *always contained a target*; these were the catch trials. The addition of the catch trials raised the overall target prevalence rate to 44%. The fake targets looked identical to the real targets (i.e. the fake targets were also rotated letter T’s). Observers were informed at the start of the experiment that these fake target trials would be included and that feedback would be provided only for these fake target trials. Thus, observers were made fully aware that a large number of additional trials had been included specifically for the purpose of increasing the overall prevalence rate. This was done because in a clinical setting we would not be able to deceive professional radiologists. So to simulate this, we explicitly informed our participants of the purpose of the experiment.

The observers responded to the images in the CT condition in exactly the same way that they responded to the images in the DS condition. If they saw a target (i.e. a rotated letter T) they clicked on it. If they couldn’t find a target, they would click on the words “target absent” at the top of the screen. As before, participants were asked to confirm their response after each trial and were given the option to change their response.

The reviewers were assigned into 10 pairs where the first reviewer in each pair viewed all the *non-catch trials* ringed by the observers as target present. The second reviewer in each pair only viewed the trials rejected by the first reviewer as target absent. No feedback was provided to the reviewers. The hit rate and false alarm rate was calculated based only on the responses to the test images, not to the responses for the catch trial images as the responses to catch trial images were not reviewed by the reviewers.

### Results

On average the initial observers (i.e. not the reviewers) changed their response on 2.8% of the trials across the two conditions. There was no significant difference in the proportion of trials in which observers changed their mind in the DS and CT conditions, *t*(25) = 0.44, *p* = .66. Mean response time (standard error of the mean in brackets) for the observers to respond target present was 9.00 (1.44) seconds in the DS condition and 7.83 (0.64) seconds in the CT condition, the difference in mean response time was not significant, *t*(25) = 0.72, *p* = .48. The average time for observers to respond target absent in the DS condition was 13.5 (1.54) seconds, significantly faster than that in the CT condition of 22.8 (2.11) seconds, *t*(25) = 3.60, *p* = .001.

**Fig 2:**
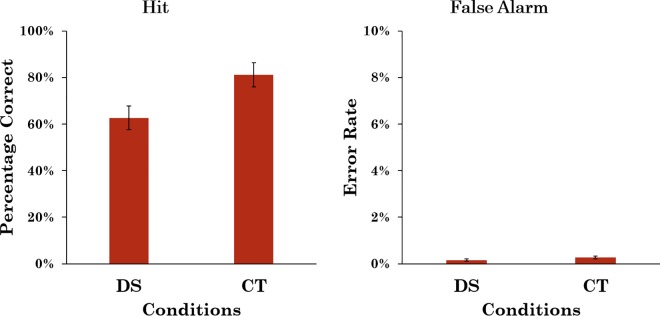
Average hit and false-alarm rates for Experiment 1. Error bars represent 95% confidence intervals.

The hit rate in the DS condition was calculated as the proportion of target-present trials on which both observers or at least one observer and the reviewer responded “present”. This was in accordance with the current practice of Breast Screen Victoria. The false alarm rate was calculated as the proportion of target-absent trials where both observers or at least one observer and the reviewer responded “present”. The hit rate in the CT condition was computed only for the test images (i.e. not for any of the catch trial images as the responses to the catch trial images were not reviewed). It was computed as the proportion of target-present trials on which at least one of the reviewers agreed with the observer that a target was present, and the false alarm rate was computed using the same criteria but using the target-absent trials. The average hit rate and false alarm rate for both conditions are shown in Fig 2. A bootstrap analysis [21] revealed that the odds ratio that hit rate was higher in the CT condition than in the DS condition was 9.06:1 and that the odds ratio that the false alarm rate was higher in the CT condition than in the DS condition was 5.99:1.

**Fig 3:**
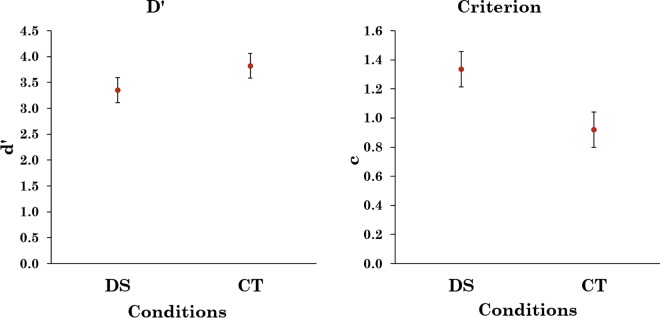
d’ and c for Experiment 1. Error bars represent 95% confidence intervals.

Fig 3 shows the mean value for the signal detection parameters, *d’* as a measure of sensitivity and *c* as a measure of response bias. Because these measures rely on the false alarm rates being non-zero, half an incorrect response was added where a participant made no false alarms [22]. Statistical analysis was also conducted using non-parametric measures of sensitivity, specifically *A* and *b* [23], and the patterns of results were very similar. Consequently, here we report only *d’* and c. A bootstrap analysis revealed that the odds ratio that *d’* was higher in the CT condition than that in the DS condition was 3.24:1. The odds ratio that *c* was smaller in the CT condition compared to the DS condition was 11.36:1, suggesting that in the CT condition participants had a more liberal criterion, so were more likely to respond ‘target present’ as the prevalence rate increased, consistent with reports in previous studies [10, 12, 17].

### Discussion

There is evidence that in this experiment increasing the apparent prevalence of the targets increased the hit rate but may also have increased the false alarm rate. So while it appears that there has been a criterion shift the evidence that sensitivity has also increased is less clear. This experiment used classic visual search stimuli. Studies that used realistic stimuli have found that increasing the apparent prevalence rate results in a greater increase in *d’* compared to what we have found (e.g. [10]). Consequently, Experiment 2 sought to repeat Experiment 1 using stimuli that appeared more similar to real tumours in mammograms.

## Experiment 2: More Realistic Stimuli

Abnormalities in mammograms can very roughly be classified into three broad categories based on appearance: circumscribed, stellate, and calcifications. A circumscribed tumour has the shape of a semi-transparent oval, a stellate tumour appears spiky and calcifications look like bright dots on a mammogram. Targets in this experiment were white semi-transparent ovals with fuzzy boundaries representing malignant circumscribed tumours. Distractors were semi-transparent ovals with well-defined boundary, designed to approximately simulate the appearance of benign circumscribe tumours. Our procedure was otherwise identical to the previous experiment.

### Method

#### Participants

Thirty-four participants between the ages of 19 and 29 years participated in this experiment (*M* age = 23.24 years, *SD* = 2.54 years; 20 women). 14 participants were tested in pairs as observers in the DS condition and 13 of these observers were also tested as observers in the CT condition, in a counter-balanced fashion. Twenty participants were tested in pairs as the reviewers in the CT condition, and individually as reviewers in the DS condition, again in a counterbalanced fashion.

#### Materials & Procedure

**Fig 4:**
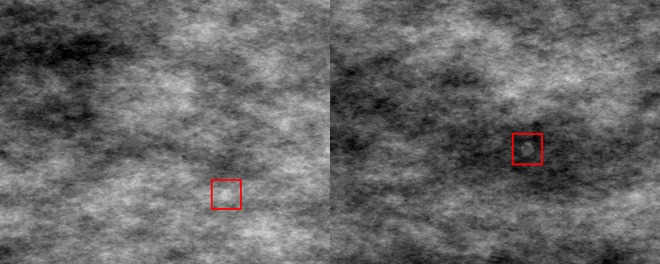
Sample stimulus of a target trial (left) and a distractor trial (right) from Experiment 2.

The stimuli were white semi-transparent (opacity = 30%, luminance = 20.8 cd/m^2^) circles with a radius of 0.29° of visual angle. The target was a disk with a fuzzy boundary, simulating the appearance of a malignant circumscribed tumour on a mammogram. The distractor was a disk with a clear boundary, simulating a benign tumour. Because participants were not familiar with the targets, to equate the level of expertise between observers and reviewers, all reviewers were given a training session of 233 trials with a prevalence rate of 44% prior to the commencement of the actual experiment. The rest of the materials and procedure were identical to Experiment 1. An example of the stimulus is shown in Fig 4.

### Results

On average, participants changed their response on 4.7% of the trials across the two conditions. The difference was not significant, *t*(25) = 1.84, *p* = .077. Mean response time (SEM in brackets) for answering “target present” was 17.6 (2.1) seconds in the DS condition and 9.65 (1.5) seconds in the CT condition. This difference turned out to be statistically significant, *t*(25) = 2.98, *p* = .006, Cohen’s *d* = 1.19. The average response time for answering “target absent” was 15.2 (2.1) seconds in the DS condition and 14.9 (1.2) seconds in the CT condition. This difference was not statistically significant, *t*(25) = 0.11, *p* = .91.

**Fig 5:**
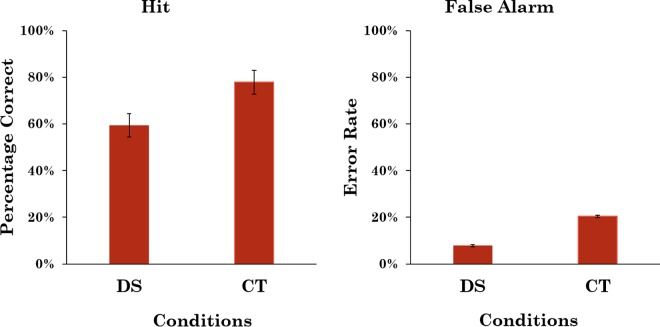
Average hit and false-alarm rates for Experiment 2. Error bars represent 95% confidence intervals.

Hit rate and false alarm were calculated in the same manner as in Experiment 1 and shown in Fig 5. A bootstrap analysis [21] revealed that the odds ratio for hit rate to be higher in the CT condition compared to the DS condition was 8.94:1 and the odds ratio that the false alarm rate was higher in the CT condition than that in the DS condition was over 1000:1.

**Fig 6:**
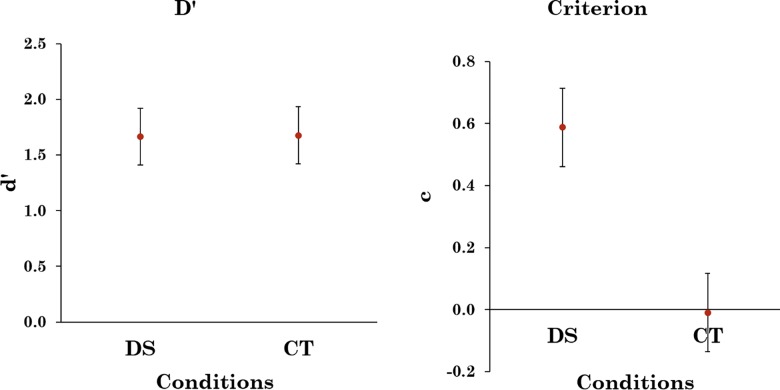
d’ and c for average hit rate and FA in Experiment 1, error bars represent 95% confidence intervals.

Fig 6 shows the signal detection parameters for Experiment 2, again, non-parametric measurements of sensitivity revealed the same pattern of results as *d’* and *c*. Unlike that in the previous experiment, there was no indication of an increase in sensitivity in the CT condition compared to the DS condition. A bootstrap analysis [21] found that the odds ratio that *d’* was higher in the CT condition than the DS condition was 0.85:1 and the odds ratio for *c* being more liberal in the CT condition than the DS condition was 11.36:1. This suggests that changing the apparent prevalence changed the criterion more than the sensitivity.

### Discussion

Similar to the results in Experiment 1, participants found more targets in the high prevalence CT condition and were more inclined towards a target-present response. However, false alarm increased almost three-fold in the current experiment and this prevented an increase in sensitivity. If our aim was to simply propose an alternative to the double screening protocol while improving, or at least maintaining, the hit rate, then inserting target present trials to boost up overall prevalence rate proved to be an effective way of achieving this aim. Nonetheless, increasing target detection at the expense of a 20% false alarm rate would be too costly for cancer screening institutes to implement. Hence in practical terms our proposed intervention was not a success.

## General Discussion

The aim of our study was to find a way to improve tumour detection in mammograms. Because the probability of a cancer being present in any given mammogram is very low, radiologists often miss tumours when they are present [8, 9], an issue known as the prevalence effect [6]. In an attempt to reduce the prevalence effect, we introduced fake target trials into a low prevalence search task that was designed to simulate breast screening. The introduction of the fake targets increased the apparent prevalence of the targets. In the first experiment the results were ambiguous. In that experiment we found that introducing fake targets led to a more liberal observer response bias, or criterion, and this in turn increased the hit rate as has been observed in prior studies [4–6, 12, 14, 16]. However, because the targets were easily distinguishable from the distractors (i.e. the background noise) this criterion shift in CT did not increase the false alarm rate to the same extent and, as a result, there was some evidence for an increase in sensitivity (*d’*). In other words, when observers are good at recognising the targets (i.e. the false alarm rate is low), our data suggests that introducing fake targets may be an effective way of reducing the prevalence effect.

In our second experiment, our results were less promising. In this experiment, false alarm rates in the condition that represents the standard Breast Screen Victoria protocol (the DS condition) was not negligible. This occurred primarily because in this experiment, noise in the background was much more similar to the targets and could not always be reliably distinguished from them. As before, introducing fake targets in the CT condition caused observers to adopt a more liberal criterion, increasing the hit rate. However, unlike the previously experiment, this liberal criterion also increased the false alarm rate. The net result was that sensitivity remained essentially unchanged. Thus for situations where targets cannot always be distinguished from distractors or the background, introducing fake targets does not seem to be a good cure for the prevalence effect.

In an attempt to explain the prevalence effect, Wolfe and Van Wert [24]developed a two-stage model. In the first stage of this model, the visual system selects each item in a search array in turn and gathers information to determine whether the item selected is the target. As prevalence decreases, the decision criterion for target-presence becomes more conservative; more evidence is required to identify an item as the target and the system is more biased towards answering “no”. As a result, the likelihood of missing a target increases. On the other hand, as prevalence increases, the system is more biased towards answering “yes” and leads to increased rate of false alarms. The second stage of the model consists of a quitting threshold that determines when search should be terminated [12, 25]. Lower prevalence rate lowers this threshold and observers terminate the search more rapidly, whereas higher prevalence rate shifts up this threshold and results in a slower target-absent response. This explains why RTs for target-present responses are typically not influenced by a change in the prevalence rate [17]. Data from our experiments generally supported the first stage of this model. In both of our experiments we observed an increase in both hit rate and false alarm rate, and the criterion was more liberal in the high prevalence CT condition. However, part of our findings were inconsistent with the prediction of the second stage of the model. When participants were good at distinguishing the target from the background (as evidenced by low false alarm rates), as was the case in Experiment 1, the time they took to respond “target present” was not affected by the prevalence rate, as predicted by the model. But when the target was easily confusable with the background, as was the case in Experiment 2, participants responded “target present” faster when the prevalence rate was high, contrary to the predictions of the model. The “target absent” response times are also partially inconsistent with this model. Reducing target prevalence decreased “target absent” response times only in Experiment 1. Inconsistent with the model, prevalence rate had no effect on “target absent” response times in Experiment 2. While the model of Wolfe and Van Wert appears to provide a good account of findings in Experiment 1, it does not appear to be as consistent with the data from Experiment 2.

In summary, our intervention was only a partial success. Introducing fake targets increased the hit rate in simulated breast cancer screening tasks. When the targets could easily be distinguished from the distractors and the noisy background (as evidenced by low false alarm rates), there was some weak evidence that sensitivity improved as the hit rate improved. However, when the targets could not always be easily distinguished from the noisy background, inserting fake targets increased both the hit rate and false alarm rate, and the observer’s sensitivity remained approximately the same. We acknowledge that the participants in our study were mainly composed of undergraduate students who lacked any training in mammography. Conversely, breast cancer screening is routinely done by trained radiologists with extensive experience. While it is possible that professional radiologists could potentially benefit from the insertion of catch trials more than our undergraduate students, there is no a priori reason to expect this to be the case. In particular, in real mammograms it is sometimes very difficult to identify a malignant tumour as evidenced by false alarm rates on the order of 20% [8]. Since the false alarm rate in our experiments was always similar to or less than this level, there is no evidence that the undergraduates were less adept at recognising simulated tumours than real radiologists are adept at recognising real tumours. Consequently, given the failure of our intervention to work for our simulated mammograms, there is no reason to believe that it would work for real mammograms, so we cannot recommend that the CT protocol be trialled in a clinical setting.

## Supporting Information

S1 File ChenHowe2016_data.xlsx. (XLSX)

## References

[1] Bech AG. Breast Cancer in Australia: An Overview: AIHW; 2012.

[2] Godwin HJ, Menneer T, Cave KR, Helman S, Way RL, Donnelly N. The impact of relative prevalence on dual-target search for threat items from airport X-ray screening. Acta psychologica. 2010;134(1):79–84. 10.1016/j.actpsy.2009.12.009 20129597

[3] Menneer T, Donnelly N, Godwin HJ, Cave KR. High or low target prevalence increases the dual-target cost in visual search. Journal of Experimental Psychology: Applied. 2010;16(2):133 10.1037/a0019569 20565198

[4] Rich AN, Kunar MA, Van Wert MJ, Hidalgo-Sotelo B, Horowitz TS, Wolfe JM. Why do we miss rare targets? Exploring the boundaries of the low prevalence effect. Journal of Vision. 2008;8(15):15 10.1167/8.15.15 19146299 PMC3069706

[5] Wolfe JM, Horowitz TS, Van Wert MJ, Kenner NM, Place SS, Kibbi N. Low target prevalence is a stubborn source of errors in visual search tasks. Journal of Experimental Psychology: General. 2007;136(4):623 10.1037/0096-3445.136.4.623 17999575 PMC2662480

[6] Wolfe JM, Horowitz TS, Kenner NM. Cognitive psychology: rare items often missed in visual searches. Nature. 2005;435(7041):439–40. 10.1038/435439a 15917795 PMC4224304

[7] Rich AN, Hidalgo-Sotelo B, Kunar MA, Van Wert MJ, Wolfe JM, editors. What happens during search for rare targets? Eye movements in low prevalence visual search. annual meeting of the Vision Sciences Society, Sarasota, FL; 2006.

[8] Evans KK, Birdwell RL, Wolfe JM. If you don’t find it often, you often don’t find it: Why some cancers are missed in breast cancer screening. 2013 10.1371/journal.pone.0064366 23737980 PMC3667799

[9] Evans KK, Tambouret RH, Evered A. Prevalence of abnormalities influences cytologists’ error rates in screening for cervical cancer. Archives of pathology & laboratory medicine. 2011;135(12):1557 10.5858/arpa.2010-0739-OA 22129183 PMC3966132

[10] Wolfe JM, Brunelli DN, Rubinstein J, Horowitz TS. Prevalence effects in newly trained airport checkpoint screeners: Trained observers miss rare targets, too. Journal of vision. 2013;13(3). 10.1167/13.3.33 24297778 PMC3848386

[11] Fleck MS, Mitroff SR. Rare targets are rarely missed in correctable search. Psychological Science. 2007;18(11):943–7. 10.1111/j.1467-9280.2007.02006.x 17958706

[12] Kunar MA, Rich AN, Wolfe JM. Spatial and temporal separation fails to counteract the effects of low prevalence in visual search. Visual cognition. 2010;18(6):881–97. 10.1080/13506280903361988 21442052 PMC3064483

[13] Godwin HJ, Menneer T, Cave KR, Donnelly N. Dual-target search for high and low prevalence X-ray threat targets. Visual Cognition. 2010;18(10):1439–63. 10.1080/13506285.2010.500605

[14] Van Wert MJ, Horowitz TS, Wolfe JM. Even in correctable search, some types of rare targets are frequently missed. Attention, Perception, & Psychophysics. 2009;71(3):541–53. 10.3758/APP.71.3.541 19304645 PMC2701252

[15] Green D, Swets J. Signal detection theory and psychophysics: Wiley New York; 1966.

[16] Ishibashi K, Kita S. Probability cueing influences miss rate and decision criterion in visual searches. i-Perception. 2014;5(3):170 10.1068/i0649rep 25469223 PMC4249987

[17] Godwin HJ, Menneer T, Cave KR, Thaibsyah M, Donnelly N. The effects of increasing target prevalence on information processing during visual search. Psychonomic bulletin & review. 2014;22(2):469–75. 10.3758/s13423-014-0686-2 25023956

[18] Fleck MS, Samei E, Mitroff SR. Generalized “satisfaction of search”: Adverse influences on dual-target search accuracy. Journal of Experimental Psychology: Applied. 2010;16(1):60 10.1037/a0018629 20350044 PMC3653986

[19] Brainard DH. The psychophysics toolbox. Spatial vision. 1997;10:433–6. 10.1163/156856897x00357 9176952

[20] Pelli DG. The VideoToolbox software for visual psychophysics: Transforming numbers into movies. Spatial vision. 1997;10(4):437–42. 10.1163/156856897x00366 9176953

[21] Efron B, Tibshirani RJ. An introduction to the bootstrap New York: Chapman & Hall/CRC; 1998.

[22] Macmillan NA, Creelman CD. Detection theory: A user's guide: Psychology press; 2004.

[23] Zhang J, Mueller ST. A note on ROC analysis and non-parametric estimate of sensitivity. Psychometrika. 2005;70(1):203–12. 10.1007/s11336-003-1119-8

[24] Wolfe JM, Van Wert MJ. Varying target prevalence reveals two dissociable decision criteria in visual search. Current Biology. 2010;20(2):121–4. 10.1016/j.cub.2009.11.066 20079642 PMC2818748

[25] Chun MM, Wolfe JM. Just say no: How are visual searches terminated when there is no target present? Cognitive psychology. 1996;30(1):39–78. 10.1006/cogp.1996.0002 8635311

